# Summer drought alters carbon allocation to roots and root respiration in mountain grassland

**DOI:** 10.1111/nph.13146

**Published:** 2014-11-10

**Authors:** Roland Hasibeder, Lucia Fuchslueger, Andreas Richter, Michael Bahn

**Affiliations:** 1 Institute of Ecology, University of InnsbruckSternwartestraße 15, 6020, Innsbruck, Austria; 2 Division of Terrestrial Ecosystem Research, Department of Chemical Ecology and Ecosystem Research, University of ViennaAlthanstraße 14, 1090, Vienna, Austria

**Keywords:** ^13^C pulse labelling, belowground carbon allocation, carbohydrate pools, drought, osmotic adjustment, root respiration

## Abstract

- Drought affects the carbon (C) source and sink activities of plant organs, with potential consequences for belowground C allocation, a key process of the terrestrial C cycle. The responses of belowground C allocation dynamics to drought are so far poorly understood.
- We combined experimental rain exclusion with ^13^C pulse labelling in a mountain meadow to analyse the effects of summer drought on the dynamics of belowground allocation of recently assimilated C and how it is partitioned among different carbohydrate pools and root respiration.
- Severe soil moisture deficit decreased the ecosystem C uptake and the amounts and velocity of C allocated from shoots to roots. However, the proportion of recently assimilated C translocated belowground remained unaffected by drought. Reduced root respiration, reflecting reduced C demand under drought, was increasingly sustained by C reserves, whilst recent assimilates were preferentially allocated to root storage and an enlarged pool of osmotically active compounds.
- Our results indicate that under drought conditions the usage of recent photosynthates is shifted from metabolic activity to osmotic adjustment and storage compounds.

## Introduction

Meteorological extreme events such as severe summer droughts are expected to occur more frequently in a future climate (IPCC, 2012, 2013; Dai, 2011) and to exert major impacts on the carbon (C) balance of terrestrial ecosystems (Ciais *et al*., 2005; Zhao & Running, 2010; Reichstein *et al*., 2013; Bahn *et al*., 2014). Limited soil water availability impairs plant growth (Muller *et al*., 2011) and alters biomass allocation (Poorter *et al*., 2012), as well as photosynthesis and respiration of plants and ecosystems (Flexas *et al*., 2006; Atkin & Macherel, 2009; Schwalm *et al*., 2010; Pinheiro & Chaves, 2011; Selsted *et al*., 2012). Plant growth is generally more rapidly and strongly affected than photosynthesis and maintenance respiration, which results in increased tissue concentrations of nonstructural carbon (Galvez *et al*., 2011; McDowell *et al*., 2011; Muller *et al*., 2011), commonly interpreted as osmotic adjustment of plant tissues to water deficit (Chaves *et al*., 2003; Chen & Jiang, 2010). Furthermore, drought responses of growth and respiration have been suggested to be more severe in aboveground plant organs compared with those in roots (Flexas *et al*., 2005; Poorter *et al*., 2012; Sicher *et al*., 2012), but less is known about such organ-specific differences in the drought responses of carbon reserves. Moreover, it is unclear whether and how drought alters the partitioning of assimilated and belowground allocated carbon to root storage and utilisation for respiration and the growth of roots.

Belowground carbon allocation (BCA) is a crucial process within the carbon cycle of terrestrial ecosystems, coupling the activity of major source (leaves) and sink organs (roots) (Kuzyakov & Gavrichkova, 2010; Brüggemann *et al*., 2011; Epron *et al*., 2012; Bahn *et al*., 2013). Though the role of sink vs source activity in controlling BCA is still an issue of debate (Farrar & Jones, 2000; Wiley & Helliker, 2012; Palacio *et al*., 2014), BCA is likely affected by differential drought responses of above- and belowground organs. There is increasing evidence that drought slows down and reduces the translocation of recent carbon to roots and soil microbes (Ruehr *et al*., 2009; Barthel *et al*., 2011; Fuchslueger *et al*., 2014). These effects on BCA have been suggested to result from limited carbon uptake, that is, source activity, and, consequently, reduced carbon export from leaves (Ruehr *et al*., 2009; Barthel *et al*., 2011). However, it is unclear whether drought increases or decreases the belowground demand for carbon, which is considered as a key driver of BCA.

On the one hand, drought has been found to increase the proportion of fresh assimilates (relative to C uptake) allocated belowground, which has been hypothesised to result from immediate utilisation of assimilates for maintenance processes or to be associated with increased belowground C demand for root growth (Palta & Gregory, 1997; Barthel *et al*., 2011; Burri *et al*., 2014). Also an increasing requirement for osmotically active C compounds under drought (Chaves *et al*., 2003) could enhance the overall C demand of roots. On the other hand, root respiration has been commonly observed to decline under drought (Bryla *et al*., 1997; Burton *et al*., 1998; Huang & Fu, 2000; Thorne & Frank, 2008). Underlying mechanisms have been suggested to include (1) physiological adaptions to reduced growth and nutrient demand for growth (Espeleta & Eissenstat, 1998; Eissenstat *et al*., 1999), (2) direct effects of soil drying to root cell integrity (Huang *et al*., 2005), (3) limitation of respiratory substrate supply due to reduced photosynthetic carbon supply (Flexas *et al*., 2006; Atkin & Macherel, 2009). While the last hypothesised mechanism should primarily reflect reduced source activity, mechanisms (1) and (2) suggest that drought leads to a decrease in the belowground carbon demand. This clearly contrasts the previous hypothesis of enhanced belowground carbon demand under drought and highlights the considerable uncertainty in our understanding of the governing processes.

To be able to unravel the potential mechanisms underlying drought effects on BCA it is important to understand whether and how the partitioning of recently assimilated carbon to different belowground compartments and processes responds to drought. Here, we study the effects of an extreme summer drought on BCA in mountain grassland and its consequences for root carbohydrate metabolism. We address the hypotheses that: (1) drought slows down and reduces the amount but increases the proportion of recent carbon allocated belowground, and (2) drought increases the allocation of recently assimilated carbon to osmotically active compounds while reducing its allocation to root respiration. The latter hypothesis is based on the assumption that severe drought increases the demand for osmotic protection while decreasing the demand for nutrient uptake and growth. Combining experimental drought manipulation with ^13^CO_2_ pulse labelling and chasing the tracer to different above- and belowground carbohydrate pools and to root respiration, we assessed the response of BCA to drought in an intact mountain grassland.

## Materials and Methods

### Site

The study site is located at a mountain meadow (1820 m above sea level (asl)) in the Stubai valley in the Austrian Central Alps (47°7′45″N, 11°18′20″E) and is described in Bahn *et al*. ( 2009). Briefly, the site is situated at a southeast exposed and softly sloped hillside; vegetation is a *Trisetetum flavescentis* community consisting of perennial grasses and forbs; the soil is a dystric cambisol (topsoil pH = 5.5); average annual temperature is 3°C and the annual precipitation is 1100 mm. The meadow is slightly grazed in spring and autumn, mowed once in early August when the peak biomass is reached and fertilised with manure every 2–3 yr.

### Experimental set-up

Summer drought was simulated as described in Fuchslueger *et al*. ( 2014). Three rainout shelters with a base area of 3 × 3.5 m, 2.5 m height and covered by transparent and UV-B transmissive plastic foil (UV B Window; Folitec, Westerburg, Germany; light transmittance *c*. 95%) were set up for 8 wk, from 16 June until 11 August 2010. The same plots were subjected to a similar treatment in the previous year. The shelters were open at the bottom (up to 0.5 m above ground) and at the top of the face sides to allow interior ventilation. Plots of 1 × 1 m were located in the centre of each rainout shelter and corresponding control plots of the same size were sited outside *c*. 5 m off. Three days before rain exclusion was ended, the entire site was mowed and cuttings removed.

A micrometeorological station provided continuous and half-hourly logged (CR10X; Campbell Scientific, Logan, UT, USA) data on precipitation (rain gauge model 52202; R. M. Young, Traverse City, MI, USA), air temperature and humidity (HMP155 with radiation and precipitation shield DTR500; Vaisala, Helsinki, Finland) at 1.5 m above ground level, as well as soil temperature (thermocouple probe TCAV; Campbell Scientific) and soil water content (ML2x; Delta-T Devices, Cambridge, UK) at 5, 10 and 20 cm soil depth of control plots. A similar setup using different equipment for soil temperature (sensor S-TMB and data logger HOBO Micro Station H21-002; Onset Computer Corporation, Bourne, MA, USA) and soil water content (Sensor ECH2O EC-5 and data logger Em50; Decagon Devices, Pullman, WA, USA) was installed inside one of the rainout shelters. Prior installation, readings of temperature sensors were compared across sensors and proved to range within 0.5 K. Sensor-derived soil moisture data were corrected using gravimetrically measured water content of soil samples.

### Pulse labelling procedure

Pulse labelling with ^13^C was carried out in the second half of the rain exclusion treatment in three consecutive campaigns (22, 28 and 31 July 2010), each including labelling of one drought and the corresponding control plot (as in Fuchslueger *et al*., 2014). Plots were equipped with a plastic frame of 1 × 1 m and 15 cm high, inserted 3 cm into the soil several weeks before labelling. The pulse labelling procedure was similar to as described by Bahn *et al*. ( 2009, 2013). In brief, a transparent Plexiglas chamber (light transmittance *c*. 95%) of 1 × 1 × 0.7 m^3^ with a rubber gasket at the bottom edge was placed and fixed on the plastic frame to ensure gas tightness. Pressurisation of the interior was avoided by an opening at the chamber's top which was closed after fastening the chamber on the frame, and a venting tube installed in the bottom gasket. Air circulation and temperature stabilisation was achieved by fans and ice packs mounted on the shady side of the chamber, respectively. During pulse labelling we monitored the interior air temperature (shaded thermocouple), CO_2_ concentration (EGM-4 and GMP343; PP Systems, Hitchin, UK, and Vaisala, Helsinki, Finland, respectively) and the ^13^C isotope ratio of CO_2_ (QCLAS-ISO; Aerodyne Research, Billerica, MA, USA). Irradiance was measured outside the chamber using a PAR quantum sensor (PQS 1; Kipp & Zonen, Delft, the Netherlands). Pulse labellings were carried out on clear days between 9:30–13:00 h CET. After closing the chamber and once the interior CO_2_ concentration dropped to *c*. 250 ppm, highly enriched (99.9 atom-%) ^13^CO_2_ was added at flow rates of 10–40 ml min^−1^. Thereby, we established and maintained CO_2_ concentrations between 450–650 ppm and achieved ^13^C concentrations of interior CO_2_ in the range 18–24 atom-% towards the end of the procedure (for more details see Fuchslueger *et al*., 2014; Supporting Information Table S1). Each pulse labelling lasted for 90 min.

### Sampling

Plant samples were harvested in the first week of rain exclusion, immediately before and after pulse labelling, 2 and 4 h, and 1, 2, 4 and 8 d afterwards. Each sample consisted of two soil blocks including aboveground plant parts (5 × 7 cm^2^, 10 cm in depth), which were pooled to increase representativeness. Shoots were abscised, immediately treated by microwave (Popp *et al*., 1996) and stored in dry ice until further preparation for analyses. Roots were carefully washed from soil and mechanically damaged, obviously dead roots, and coarse roots (diameter >2 mm) were removed. From each fine root sample a subsample was treated in the same way as the shoots for analyses, while the remainder was kept moist using wet paper towel to prevent drying and was subsequently used for root respiration measurements (see next section).

The total aboveground phytomass was obtained on the basis of oven-dried plant material harvested in all plots at the time of mowing (3 d before the end of rain exclusion treatment) and corrected for gaps in the canopy resulting from previous sampling. The total root mass in a 0–10 cm soil depth was estimated by samples (5 × 5 cm^2^) taken after the end of the treatments.

### Root respiration measurements

Root respiration was measured in the field on fine root subsamples (2–4 g fresh mass), starting 40–60 min after sampling. Similar to Bahn *et al*. ( 2006), we used an IRGA-based photosynthesis measurement system (CIRAS-1 with conifer chamber PLC(5); PP Systems, Hitchin, UK). Respiration rates were determined at near-atmospheric CO_2_ concentrations (380–410 ppm) and at high air humidity (60–80% relative humidity) to prevent the roots from drying while minimizing the risk of condensation. A drying column filled with anhydrous calcium sulphate was inserted upstream from analyser to minimise the potential effects of water vapour and CO_2_ cross-sensitivity of the instrument. Placing the chamber inside a cool box filled with ice packs allowed the temperature response of root respiration between 10–20°C to be obtained. After respiration measurements, the root samples were stored cool until returned from the field (at most 8 h) and subsequently oven-dried (10 d at 60°C) to obtain the dry mass. Respiration rates were computed per unit dry mass as for an open system. The temperature responses of the root respiration rates were expressed as Q_10_, calculated from OLS linear regression of log-transformed respiration rates and temperature within 10–20°C. Measurements showing weak or nonsignificant temperature responses (i.e. *R*^2^ < 0.95 or *P *>* *0.05; six of 54 measurements) were excluded from the Q_10_ calculation.

An additional subsample of fine roots (2–4 g fresh mass) was incubated in an Erlenmeyer flask, gas-tight sealed by a rubber stopper, to obtain ^13^C concentrations in root respired CO_2_. Immediately after closing the flask and 5, 10, 15 and 30 min afterwards, air samples of 15 ml were drawn, and volumetrically replaced by CO_2_-free synthetic air, using a 0.8 mm syringe. Before each gas sampling, the syringe was flushed with CO_2_-free synthetic air and the flask was gently shaken to homogenise interior air. Gas samples were injected into pre-evacuated, rubber septum sealed 12 ml tubes (Exetainer; Labco, High Wycombe, UK). In order to prevent ambient air from entering the exetainers, the gas samples were pressurised by injecting a volume of 15 ml into 12 ml tubes (Hardie *et al*., 2010). The CO_2_ concentrations and isotopic compositions of the gas samples were measured within 1 month after sampling on a Finnigan Delta V Advantage Mass Spectrometer with Finnigan GasBench (Thermo Fisher Scientific, Whaltham, MA, USA). The isotope ratios of root respired CO_2_ were calculated using the Keeling Plot approach, excluding measurements with nonsignificant (*P *>* *0.05) and weak (*R*^2^ < 0.95) OLS linear regressions (four of 54 measurements). Prelabelling samples, reflecting natural abundant ^13^C concentrations, allowed the calculation of the ^13^C excess.

### Concentrations and isotope composition of carbohydrates

Samples of shoots and fine roots were dried (72 h at 60°C) and finely ground for subsequent analyses of C and ^13^C contents by an elemental analysis-isotope ratio mass spectrometry (EA-IRMS on a EA 1110; CE Instruments, Milan, Italy, coupled to a Finnigan MAT Delta Plus IRMS; Thermo Fisher Scientific), as well as for the determination of plant carbohydrate pools. Sucrose, glucose and fructose were extracted from aliquots of finely ground plant material with deionised water at 85°C for 30 min. After centrifugation, the supernatant was transferred to ion-exchange cartridges (OnGuard II H cation exchange and OnGuard II A anion exchange cartridges; Dionex, Thermo Scientific, Vienna, Austria) to remove ionic components. The resulting neutral fraction was then analysed by HPLC-IRMS (Dionex IC 3000 system, connected by a Finnigan LC IsoLink Interface to a Finnigan Delta V Advantage Mass Spectrometer; all Thermo Fisher Scientific) (Wild *et al*., 2010), on a HyperREZ XP Ca^2+^ column (Thermo Electron, Bremen, Germany) at 85°C with 0.5 ml min^−1^ of deionised water as eluent. The starch pool in the plant material was determined after enzymatic digestion with heat stable α-amylase (Göttlicher *et al*., 2006; Richter *et al*., 2009) and the resulting glucose was measured by elemental analysis-isotope ratio mass spectrometry (EA-IRMS, see above).

### Data analyses

The proportional ^13^C allocation to root respiration and root carbohydrate pools was calculated based on the amount of tracer incorporated in the total (above- and belowground) phytomass immediately after the end of pulse labelling. Overall treatment effects in the time series of root respiration rates, Q_10_, concentrations and isotope ratios in shoots and root bulk material and carbohydrate pools were tested by Friedman tests (Friedman, 1937), using paired datasets from corresponding drought and control plots. To illustrate the course of root respiration rates, Q_10_ and carbohydrate concentrations during the rain exclusion period data were pooled in time to sets measured within mostly 2 (maximum 5) d. Treatment effects within single dates of time series were tested using Wilcoxon signed-rank tests with sample sizes of *n *>* *3, and exact permutation tests (Ernst, 2004) elsewise. An exponential model according to *N*(*t*) = N_0_ e^−λ t^ was applied to the time series of ^13^C excess of root respired CO_2_ and carbohydrate pools, allowing further calculation of half-life by *t*_1/2_ = log_e_(2)/λ and the mean residence time (MRT) by MRT = t_1/2_/log_e_(2).

## Results

### Rain exclusion and soil microclimate

**Figure 1:**
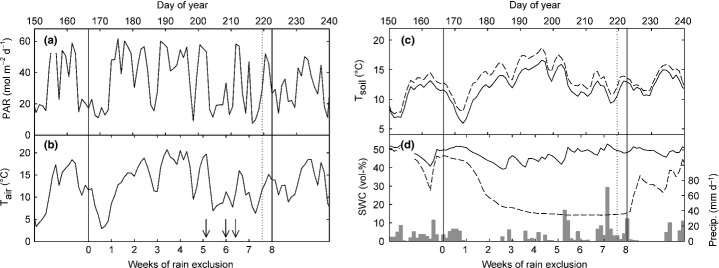
Time course of daily sums of (a) photosynthetically active radiation (PAR), (b) daily means of air temperature, and (c) of soil temperature, and (d) volumetric soil water content (SWC) in 5 cm depth, as well as daily sums of precipitation (grey bars in (d)) on the studied meadow during the summer of 2010. Dashed lines in (c) and (d) depict data from experimental drought plots. Vertical lines indicate the rain exclusion period, dotted vertical line marks the day when the site was mown. Arrows in (b) mark dates when ^13^C pulse labelling was performed.

Sheltering excluded 815 mm precipitation during 8 wk of treatment which is equal to 51% of the rainfall measured between the beginning of May (start of growing season) and the end of the treatment. Within the first half of the rain exclusion period, the daily means of soil water content (Fig. 1d) decreased to *c*. 15 vol-% (*c*. 10% of plant extractable water as estimated by the difference of field capacity and permanent wilting point based on pF curves; S. Roth *et al*., unpublished) and remained nearly constant at this level until the end of the treatment. The control plots remained moist (39–53 vol-%) throughout the experiment. The daily means of soil temperature (Fig. 1c) were increased by 1.7 K on average due to rain exclusion compared with control plots, in the range 8–19°C in drought plots vs 6–17°C in control plots, respectively.

### Carbohydrate concentrations

**Figure 2:**
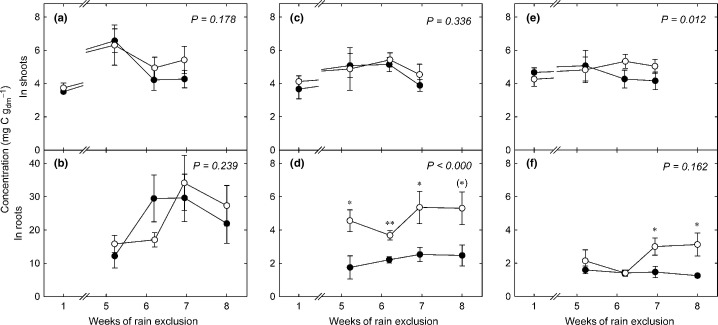
Concentrations of (a, b) starch, (c, d) sucrose, and (e, f) glucose and fructose in shoots (upper panels) and roots (bottom panels) in control plots (closed circles) and drought plots (open circles) at the beginning and towards the end of rain exclusion. Points represent mean values of time pooled samples (*n *=* *3–8) from three control and drought plots, respectively, sampled within 1–5 consecutive d. Error bars indicate ± SE. *P*-values refer to overall treatment effects, asterisks indicate significance levels of difference of means between control and treatment for individual sampling dates: **, *P *≤* *0.01; *, *P *≤* *0.05; (*), *P *≤* *0.1.

The time-pooled starch concentrations in shoots and roots (Fig. 2a,b) varied largely throughout the experiment (3.5–6.6 mg C g^−1^ in shoots and 12.2–34.1 mg C g^−1^ in roots) but remained unaffected by drought. Sucrose concentrations were not altered by drought in shoots (*P *=* *0.336), but were sharply increased in roots (Fig. 2c,d) (+ 104%, *P *<* *0.000). Glucose and fructose concentrations (Fig. 2e,f) were slightly increased in shoots (+ 10%, *P *=* *0.012) throughout the experiment, while in roots they were more than doubled during the last 2 wk of treatment compared with the controls (+ 114%, *P *=* *0.004).

### Tracer dynamics in carbohydrate pools

**Figure 3:**
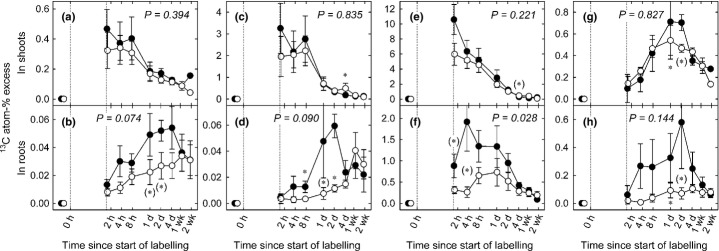
Time courses of tracer concentrations in (a, b) bulk material, (c, d) starch, (e, f) sucrose, and (g, h) in glucose and fructose of shoots and roots in control (closed circles) and drought plots (open circles). The pulse labelling period is indicated by dotted vertical lines. Error bars indicate ± SE (*n *=* *3), *P*-values refer to overall treatment effects, asterisks indicate significance levels of difference of means between control and treatment: *, *P *≤* *0.05; (*), *P *≤* *0.1.

The total incorporated ^13^C in the shoot and root phytomass, measured on samples taken immediately after the end of pulse labelling, amounted to 441 ± 153 and 271 ± 134 mg ^13^C m^−2^ (*n *=* *3, ± SE, difference *P *=* *0.250) in control and drought plots, respectively. The tracer content (atom-% excess) in shoots peaked immediately after pulse labelling in sucrose (Fig. 3e), within 8 h in starch (Fig. 3c) and bulk carbon (Fig. 3a), and 24 h after pulse labelling in glucose and fructose (Fig. 3g), irrespective of treatment. The highest initial label concentrations in shoots were found in sucrose, followed by starch.

Rain exclusion affected neither tracer concentrations (except for single dates, see Fig. 3c,e,g) nor the mean residence times (MRT) in shoot carbon pools (Table 1). The rate of tracer incorporation in roots, as reflected by the slope of concentration changes over time, was reduced by 56% (*P *=* *0.050) under drought conditions, resulting in significantly (*P *=* *0.007) lower concentrations within 1–4 d after pulse labelling and by trend lower concentrations (*P *=* *0.074) throughout the chase period of 2 wk (Fig. 3b). Similar trends were found in tracer concentrations of root starch (Fig. 3d) and monosaccharides (*P *=* *0.090 and *P *=* *0.144, respectively) (Fig. 3h), with the largest differences 2 d after pulse labelling. Tracer recovery in root sucrose (Fig. 3f) diminished strongly due to rain exclusion (*P *=* *0.028) while the MRT of tracer in this pool increased consistently (Table 1).

**Table 1:** Results of quantitative analyses of decay curves of ^13^C tracer in aboveground and belowground sucrose pools and in root respired CO_2_ of each three control and rain exclusion plots

C Pool	Treatment	Peak (h)^a^	Exponential fit^b^				MRT (h) mean ± SE	*P*-value of difference in MRT^c^
			*n*	*R*^2^	*P-*value	MRT (h)		
Shoot sucrose	Control	1.5	7	0.791	0.007	5	13 ± 5	0.400
		1.5	7	0.883	0.002	22		
		1.5	8	0.956	0.000	12		
	Rain exclusion	1.5	7	0.994	0.000	28	16 ± 7	
		1.5	7	0.804	0.006	3		
		4	7	0.972	0.000	16		
Root sucrose	Control	48	4	0.915	0.044	62	57 ± 4	0.100
		4	7	0.823	0.005	62		
		4	7	0.851	0.003	49		
	Rain exclusion	24	5	0.858	0.024	108	107 ± 1	
		8	6	0.819	0.013	105		
		8	6	0.289	0.271	–		
Root respired CO_2_	Control	24	4	0.996	0.002	108	93 ± 7	0.200
		8	6	0.981	0.000	88		
		8	7	0.903	0.001	84		
	Rain exclusion	24	5	0.949	0.005	341	239 ± 83	
		24	4	0.789	0.112	301		
		8	5	0.527	0.165	75		

a Refers to the respective peak value of ^13^C obtained in samples taken 1.5, 4, 8, 24 and 48 h after pulse labelling.

b Equation *N*(*t*) = *N*_Peak_ e^−λ t^ fitted to *n* data points including each peak value using ordinary least squares estimation. Mean residence time (MRT) = λ^−1^.

c Tested by exact permutation.

**Figure 4:**
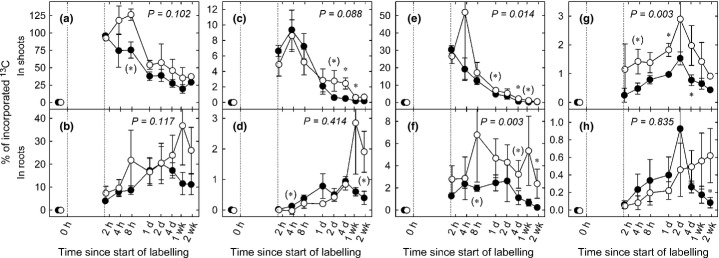
Time courses of ^13^C in shoot and root bulk material and carbohydrates relative to total incorporated ^13^C during pulse labelling (dotted vertical lines) in (a, b) bulk material, (c, d) starch, (e, f) sucrose, and (g, h) in glucose and fructose of shoots and roots. Means ± SE of each three control (closed circles) and drought (open circles) plots. *P*-values refer to overall treatment effects, asterisks indicate significance levels of difference of means between control and treatment: *, *P *≤* *0.05; (*), *P *≤* *0.1.

To assess the drought effects on the proportional allocation of recent carbon to different carbon pools we normalised the total amounts of tracer uptake during labelling (Fig. 4; note that due to within-plot variability normalised total tracer uptake exceeded 100% during the first 8 h, but this did not affect overall trends and the conclusions drawn). The proportions of tracer recovered under rain exclusion in aboveground carbon pools (Fig. 4a,c,e,g) were higher under drought conditions, particularly in sugars. In roots, a similar percentage of tracer recovered within 4 d after pulse labelling in bulk carbon, starch and hexoses, while higher proportions were observed within 1–2 wk after labelling in glucose and fructose (*P *=* *0.025) and, by trend, in starch (*P *=* *0.078) (Fig. 4b,d,h). Proportional tracer allocation to root sucrose was increased (*P *=* *0.003) by 41% on average throughout the measuring period of 2 wk (Fig. 4f).

### Root respiration and tracer dynamics in root respired CO_2_

**Figure 5:**
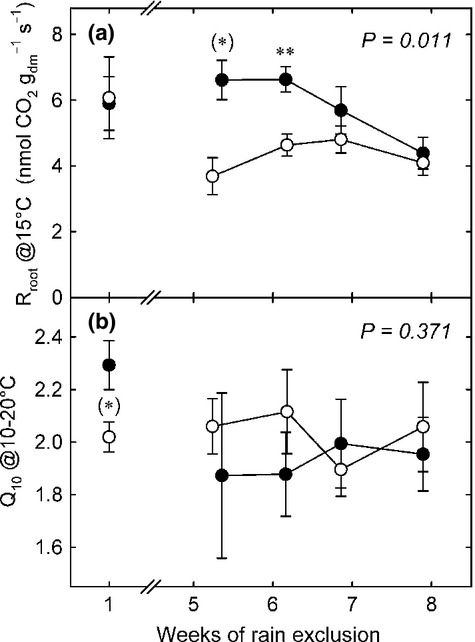
(a) Root respiration rates at 15°C reference temperature and (b) temperature coefficient of root respiration (Q_10_) between 10–20°C in control plots (closed circles) and drought plots (open circles) at the beginning and towards the end of rain exclusion. Mean values ± SE of time pooled samples (wk 1 *n *=* *3, wk 5–8 *n *=* *4–8) from three control and drought plots, respectively. *P*-values refer to overall treatment effects, asterisks indicate significance levels of difference of means between control and treatment: **, *P *≤* *0.01; (*), *P *≤* *0.1.

**Figure 6:**
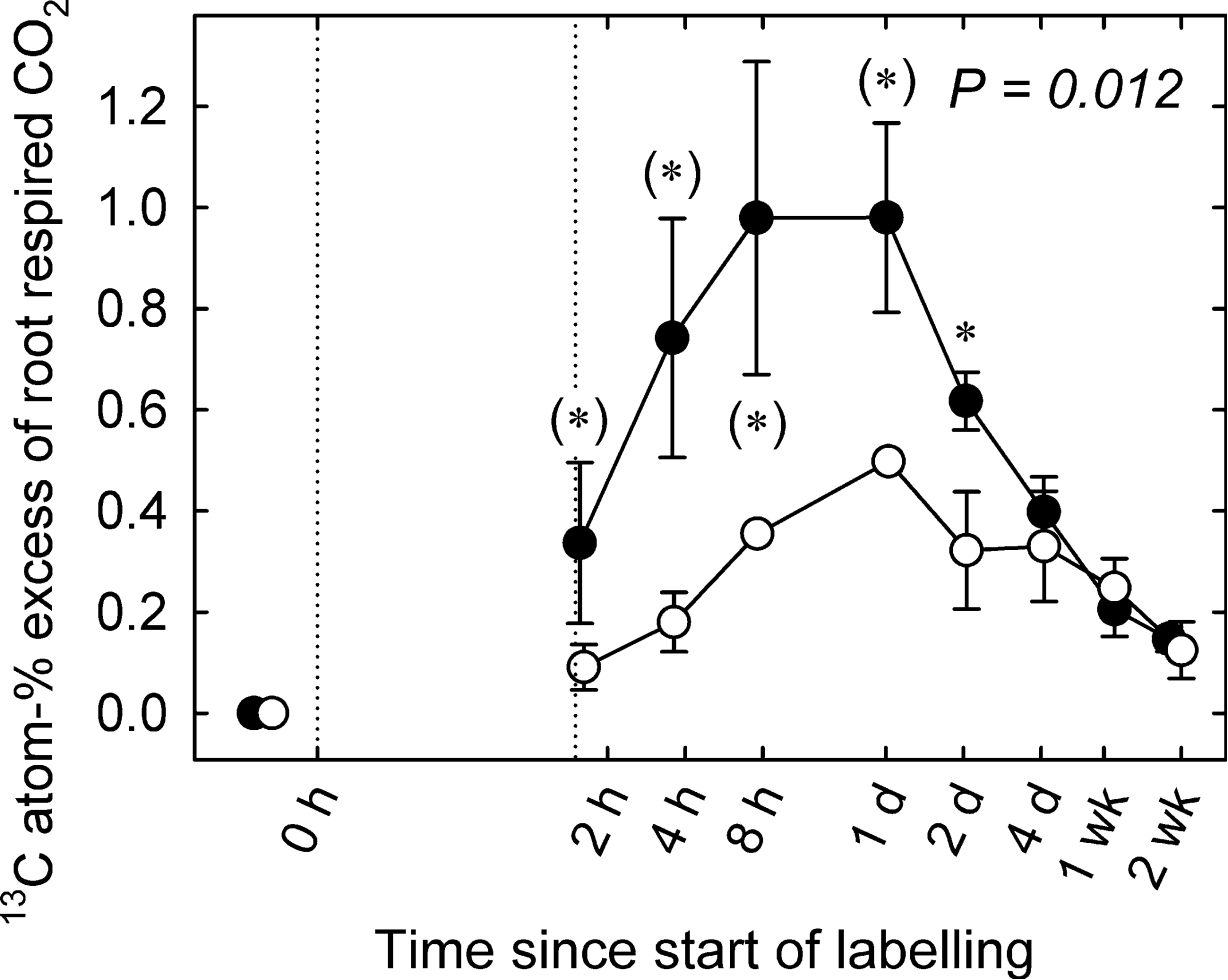
Time courses of tracer concentrations in root respired CO_2_ in control (closed circles) and drought (open circles) plots (*n *=* *3, mean ± SE). The pulse labelling period is indicated by dotted vertical lines. *P-*value refers to overall treatment effect, asterisks indicate significance levels of difference of means between control and treatment: *, *P *≤* *0.05; (*), *P *≤* *0.1.

At the beginning of the rain exclusion experiment the mean root respiration rates per g root dry mass (Fig. 5a) were similar across plots, in the range 4.3–6.6 nmol CO_2_ g^−1^ s^−1^. During the last 3 wk of rain exclusion, the mean root respiration decreased to 3.7–4.8 nmol CO_2_ g^−1^ s^−1^ which was significantly lower (*P *=* *0.002) compared with the control plots (4.3–6.6 nmol CO_2_ g^−1^ s^−1^). The temperature sensitivity of root respiration (Q_10_) remained unaffected by the drought treatment (overall average ± SE 2.0 ± 0.1) (Fig. 5b). Tracer concentrations in root respired CO_2_ (Fig. 6) peaked within 8 and 24 h after pulse labelling in control plots and after 24 h in drought plots, where peak values tended to be lower (*P *=* *0.100). The MRT of ^13^C in root respired CO_2_ (Table 1) was 93 ± 7 h in control plots and 239 ± 83 h in drought plots (*n* = 3, ± SE, *P *=* *0.200).

## Discussion

Drought reduces the carbon uptake of terrestrial ecosystems and impairs the activity of carbon sinks in plants. Sink activity and carbon demand by sinks are recognised to be a major driver of carbon allocation (Farrar & Jones, 2000; Muller *et al*., 2011; Epron *et al*., 2012; Poorter *et al*., 2012). In consequence, drought can affect the allocation of assimilates to roots. By tracing pulse-labelling-derived ^13^C in above- and belowground plant organs, carbohydrate pools and root respired CO_2_, we investigated the effects of drought on the amounts and dynamics of photosynthates allocated belowground and their partitioning amongst different sinks.

### Drought effects on amounts and dynamics of belowground carbon translocation

**Figure 7:**
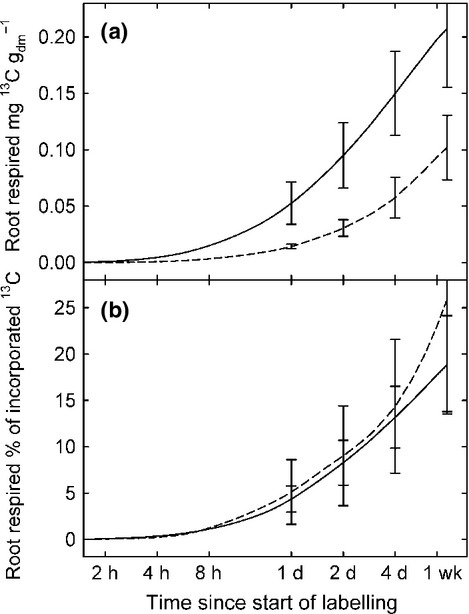
Estimation of (a) cumulative absolute amounts of tracer respired per g root mass, and of (b) cumulative percentage of tracer relative to total incorporated ^13^C after pulse labelling in control plots (solid line) and drought plots (dashed line), mean values ± SE of each three plots. Estimation is based on linearly interpolated ^13^C concentrations in root respired CO_2_ and respiration rates converted to actual soil temperatures. Labels on time axis correspond to sampling intervals.

In our study, drought reduced the amount and the speed of carbon allocation to the root biomass by *c*. 50% (Fig. 3b), thereby confirming our first hypothesis to that effect. The turnover rates of sucrose, a major compound for carbon translocation from leaves to roots (Slewinski & Braun, 2010), were lower under drought conditions in roots but not in shoots (Table 1; Fig. 3e,f). Moreover, the lower turnover rates of ^13^C in root sucrose were accompanied by increased sucrose concentrations (Fig. 2d), which resulted in a dilution effect. Thus, drought-induced impairment of BCA may not have been related to turnover rates in sugar pools. However, the reduced metabolic activity of the roots, as indicated by lower respiration rates (Fig. 5a), is likely to reflect a decreased carbon demand by the roots. Consistently, less of the recent assimilates were respired by roots under drought (Fig. 7a). In addition, the carbon allocation to rhizosphere microbes was reduced due to rain exclusion in our experiment (Fuchslueger *et al*., 2014), which further decreased the belowground carbon demand beyond that of plant metabolism. Thus, the observed decline in BCA under drought was possibly related to diminished C demand by the roots.

By contrast, a recent drought experiment in a lowland grassland did not indicate significant reductions in the amounts or in the velocity of belowground allocated carbon (Burri *et al*., 2014), which the authors interpreted as a consequence of an observed reduction in photosynthesis paralleled by a hypothesised increase in the demand of recent assimilates for root growth. However, this assumption is in line with our findings inasmuch as, in intact grassland, drought responses of BCA are significantly affected by belowground carbon sink activity. Similar responses of BCA to soil drying to those we found in our study were reported for saplings of deciduous tree species (Ruehr *et al*., 2009; Barthel *et al*., 2011) and for crops (Palta & Gregory, 1997; Salmon *et al*., 2014). These responses were associated with comparable declines in photosynthesis and turnover rates of water-soluble leaf carbohydrates and have thus been suggested to result from reduced leaf carbon export and subsequent phloem loading (Ruehr *et al*., 2009; Barthel *et al*., 2011).

A number of earlier studies have suggested that the proportional allocation of recent C to belowground (i.e. the amount of tracer allocated relative to the total amount of tracer taken up during labelling) increases under drought (Palta & Gregory, 1997; Huang & Fu, 2000; Barthel *et al*., 2011; Sanaullah *et al*., 2012; Burri *et al*., 2014). While our study, contrary to what we hypothesised, did not indicate a significant increase in the proportional BCA under drought, it showed that proportionally more tracer was incorporated in shoot and root sugars (Fig. 4e–h). This points to a decreased usage of recent carbon for aboveground growth (Poorter *et al*., 2012) and respiration (Flexas *et al*., 2005). Interestingly, carbon reserves in shoots (apart from marginally elevated glucose and fructose concentrations) did not increase under such conditions of limited aboveground carbon demand (Fig. 2a,c,e) whilst the proportion of tracer recovered in root sucrose increased (Fig. 4f). This indicates that under drought conditions grassland perennials allocate C preferentially to roots rather than to shoots, including shoot storage pools. This conclusion is underpinned by the outcome of a previous experiment conducted at the same grassland site (Bahn *et al*., 2013) showing that under severe limitation of C uptake due to extended shading BCA is maintained even at the expense of aboveground storage pools.

### Drought effects on carbon partitioning to root carbohydrate pools

Drought tended to increase the relative amount of tracer in root starch within 2 wk after pulse labelling (Fig. 4d) which hints at increased allocation of recent assimilates to root storage. In fact, there is growing evidence for preferred C partitioning to storage pools under adverse and potentially limiting environmental conditions (Sala *et al*., 2012; Dietze *et al*., 2014). Moreover, under drought conditions root storage pools were suggested to provide carbon for the accumulation of osmotically active compounds in roots (Galvez *et al*., 2011, but see next paragraph).

The proportional tracer allocation to root sucrose was more than doubled under drought conditions (Fig. 4f). Additionally, sucrose concentrations in the roots strongly increased (Fig. 2d) which is likely related to osmotic adjustments of the roots (Chaves *et al*., 2003; Chen & Jiang, 2010; Sicher *et al*., 2012). Taken together, this finding indicates that drought shifts the allocation of recent assimilates in favour of osmolyte pools. In leaves, such osmotically active compounds have been shown to originate mainly from starch degradation (Chaves *et al*., 2003; Lee *et al*., 2008), and the same mechanism was suggested to underlie the osmotic adjustment in roots of drought-stressed tree seedlings (Galvez *et al*., 2011). However, our data indicate that drought increases the partitioning of recent assimilates to osmotic adjustment in roots and that starch breakdown is unlikely to contribute to this drought-induced increase of root sugar concentrations.

### Drought effects on carbon supply of root respiration

Root respiration is a major carbon flux in terrestrial ecosystems and is known to decrease in dry soils (Lambers *et al*., 2005). This response has been suggested to be related to reduced root growth, impaired root cell integrity and limited substrate supply (Atkin & Macherel, 2009). We hypothesised that drought reduces the amount of recent assimilates allocated to root respiration resulting from the lower demand for nutrient uptake and growth. In our experiment, drought decreased root respiration by up to 44% (26% on average), which is in line with earlier observations (reviewed by Atkin & Macherel, 2009). Interestingly, the supply of root respiration with recent assimilates, as indicated by tracer allocation, was halved under drought (Figs 6, 7a). Since respiration rates decreased less compared with the decline in the allocation of fresh assimilates, respiratory substrate supply might have been balanced by an increased contribution of storage pools (Lehmeier *et al*., 2010; Hopkins *et al*., 2013; Lynch *et al*., 2013). This assumption is confirmed by the ratio between the MRT of root respiratory substrate (as reflected by tracer dynamics in root respired CO_2_; Schnyder *et al*., 2012) and the MRT of root sucrose (which is assumed to act as a major precursor of respiratory substrate in roots; Ghashghaie *et al*., 2003). In control plots, the MRT of the root respiratory substrate was higher than the MRT of root sucrose alone by a factor of 1.6 (Table 1). This indicates that a storage pool with a slower carbon turnover than sucrose contributed substantially to the overall root respiration under control conditions (Lehmeier *et al*., 2008, 2010). Under drought this ratio (MRT of root respiratory substrate over the MRT of sucrose) increased to 2.2, reflecting a shift in the substrate supply for root respiration towards increasing delivery of C from storage pools other than sucrose.

A slightly elevated soil temperature in our rain exclusion treatment (< 2 K; Fig. 1c) could have led to thermal acclimation of root respiration (Atkin & Tjoelker, 2003), potentially resulting in an overestimation of the drought effects on root respiration at the soil temperature compared with that at a reference temperature of 15°C during measurements (Atkin *et al*., 2005). Since the Q_10_ of root respiration (Fig. 5b) remained unaffected we can rule out interfering temperature effects on the observed drought responses of root respiration. To separate other factors that could have affected the observed changes in root respiration from the effects of dehydration, we measured root respiration on moist root samples. Potential effects of dehydration on root respiration rates were verified by additional measurements and revealed a decline by 24% at a relative water content of 50% compared with water saturated roots (Fig. S1). Respiration rates of moist roots (Fig. 5a) in control plots were in the range 4.3–6.6 nmol CO_2_ g^−1^ s^−1^ which is comparable to the results from earlier studies carried out at the same site (Bahn *et al*., 2006) and at other temperate grasslands (Fitter *et al*., 1998; Wang *et al*., 2010).

### Conclusions

From our study we conclude that in mountain grassland drought reduces the amount and speed of recent C allocated belowground, and increases the allocation of this C to sugar and starch reserves in roots at the expense of root respiration. This indicates a drought-induced shift in the usage of recent photosynthates from metabolic activity to storage pools, which underpins a slowing of ecosystem carbon cycling under drought. This slowing is also reflected by a reduced transfer of recent plant-derived C to root herbivores (Seeber *et al*., 2012), soil microbial communities (Fuchslueger *et al*., 2014) and soil-respired CO_2_ (Burri *et al*., 2014). However, in view of the substantial reductions in ecosystem carbon uptake under drought, immediate ecosystem carbon storage *per se* is not increased, but generally decreased under extreme drought (Reichstein *et al*., 2013). From a plant's perspective, the strategy of investing C into storage pools under unfavourable conditions and remobilisation under improved conditions enables perennial grasses and forbs to regrow rapidly and effectively after periods of severe drought, increasing the resilience capacity of grassland under extreme drought conditions.

## Supporting Information

Fig S1 Response of root respiration rates to root water content of fine roots from a mountain meadow, obtained from seven root samples measured at 4–7 different water content levels.
