# Supplementary material for: Summer drought alters carbon allocation to roots and root respiration in mountain grassland
Source: New Phytol. 2014 Nov 10;205(3):1117–27. doi: 10.1111/nph.13146 (PMC4303983; doi:10.1111/nph.13146)
Supplement: Fig S1 — Response of root respiration rates to root water content of fine roots from a mountain meadow, obtained from seven root samples measured at 4–7 different water content levels. [file nph0205-1117-sd1.pdf]

Supporting Information Fig. S1

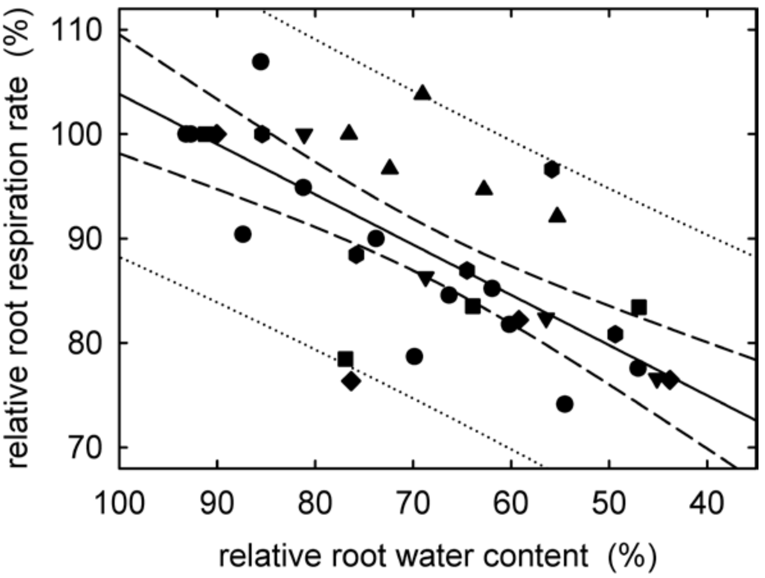

Fig. S1 Response of root respiration rates to root water content of fine roots from a mountain meadow, obtained from seven root samples (represented by different symbols) measured at 4–7 different water content levels (35 measurements in total). The fitted linear regression line (solid, $R^2 = 0.531$, $P < 0.001$) is shown together with the 95% confidence interval (dashed lines) and the 95% prediction interval (dotted lines).
